# Epidemiological, histopathological and clinical profile of breast cancer in Eastern Morocco: data from the Taza RHRC (2016–2024)

**DOI:** 10.3332/ecancer.2026.2122

**Published:** 2026-05-07

**Authors:** Nacer Bakloul, Fouzia Elaissaoui, Khalil Hammani, El Hassan Elharchli

**Affiliations:** 1 Laboratory of Natural Resources and Environment, Faculty of Taza, Sidi Mohammed Ben Abdellah University, Fes 30000, Morocco; 2 Higher Institute of Nursing Professions and Health Techniques of Taza, Opposite Moulay Rachid Middle School, Ourida, Taza 35000, Morocco

**Keywords:** breast cancer, epidemiology, histopathology, clinical

## Abstract

**Background:**

Breast cancer (BC) represents a major public health issue in Morocco. This study aims to describe the epidemiological, clinical and histopathological profile of women with BC diagnosed at the Taza Reproductive Health Reference Center in eastern Morocco between November 2016 and December 2024.

**Methods:**

This was a descriptive, retrospective, cross-sectional study of 412 confirmed cases of BC. Data were collected from archived medical records and analysed using IBM SPSS Statistics software.

**Results:**

412 new cases were identified, with an increasing trend over the years, from 15.1 per 100,000 in 2017 to 29.2 per 100,000 in 2024. The mean age at diagnosis was 51.5 ± 11.8 years. The majority of patients (59%) were aged between 40 and 59, 70.9% were married and 52.6% lived in rural areas. Most were housewives (97.3%). Regarding health coverage, 73.5% were insured, including 40.3% affiliated with RAMED’s health insurance regime. Clinically, the left breast was affected in 54.1% of cases and the predominant tumour localisation was the superolateral quadrant (57.8%). The most frequent histological type was invasive ductal carcinoma (95%). According to Scarff–Bloom–Richardson (SBR) histoprognostic grade, 68% of tumours were grade II, 28% grade III and 4% grade I.

**Conclusion:**

This study highlights a progressive increase in the incidence of SBR in the Taza region, as well as a socio-economic profile marked by precariousness and rurality. Intermediate-grade infiltrating ductal carcinoma is the dominant histological form. These results underline the importance of early detection and improved access to care, particularly in rural areas.

## Background

Over the last few decades, breast cancer (BC) has become an emblematic disease for global health inequalities [1]. More than just a disease, it represents a real challenge in terms of early diagnosis, equitable access to care and effective prevention [2]. The global distribution of this disease reveals striking contrasts: while high-income countries are recording better survival thanks to organised screening and innovative treatments, low- and middle-income countries continue to face worrying mortality rates, often linked to late diagnosis and limited access to specialist care [3, 4].

In Morocco, BC is a major public health issue. It is the most frequently diagnosed cancer in women and the leading cause of cancer-related death in this population [5]. In 2022, BC accounted for approximately 38.8% of all cancers among women in Morocco, with an estimated age-standardised incidence rate (ASR, world) of 58.4 cases per 100,000 women [6]. Data from the Greater Casablanca Cancer Registry similarly report a high burden of BC, with an ASR, world of 45.5 per 100,000 women over the 2018–2021 period [7].

In this context, several studies have examined the clinical, epidemiological and histopathological characteristics of BC in different regions of Morocco [8–10]. However, few have specifically addressed regional particularities, especially in eastern areas such as the province of Taza, where local health and social conditions may shape both the presentation of the disease and access to care. To help fill this gap, the present study provides a descriptive analysis of the epidemiological, clinical and histopathological profile of BC cases diagnosed at the Reproductive Health Reference Center (RHRC). This center functions as a second-level referral facility within the Moroccan healthcare system, receiving women referred from primary health centers for advanced diagnostic evaluation and specialised reproductive and breast health services. Covering an 8-year period from November 2016 to December 2024, this study aims to strengthen understanding of BC at the local level and to support the development of prevention, early detection and management strategies tailored to the regional context.

## Methods

### Study population

This was a descriptive, retrospective, cross-sectional study aimed at analysing the epidemiological and histopathological characteristics of BC in women treated at the RHRC of Taza, located in eastern Morocco. The study covered an 8-year period, from November 2016 to December 2024.

The study population comprised 412 patients with a confirmed diagnosis of BC at the RHRC during the study period. The inclusion criteria were all women diagnosed with BC based on pathological examination, regardless of affiliation to a medical insurance scheme (CNOPS, CNSS, RAMED or AMO-Tadamon). The exclusion criteria were: patients without a confirmed pathological diagnosis, cases of male BC and cases considered unusable or managed exclusively in the private sector. Data were collected retrospectively from medical records archived at the RHRC. Information extracted included socio-demographic, clinical and histopathological characteristics. Early-onset breast cancer (EOBC) was defined as a diagnosis made before the age of 40 years, in accordance with epidemiological standards.

From an ethical perspective, the study was conducted in accordance with the regulations in force within the institution. Authorisation for data collection was obtained from the relevant authorities and patient anonymity and confidentiality were strictly maintained throughout the research process.

### Statistical analysis

Data analysis was performed using IBM SPSS Statistics software. Descriptive statistics were used to summarise socio-demographic, clinical and histopathological characteristics, continuous variables were reported as mean ± standard deviation and range, and categorical variables as frequencies and percentages.

Associations between categorical variables were assessed using the chi-square test of independence. When expected cell counts were <5, an exact test was applied (Fisher’s exact test for 2 × 2 tables and the Fisher–Freeman–Halton exact test for R × C tables). A subgroup analysiscompared histological grade distribution between EOBC (<40 years) and non-EOBC (≥40 years). A two-sided *p*-value <0.05 was considered statistically significant.

## Results

### Incidence of BC

**Figure 1.**
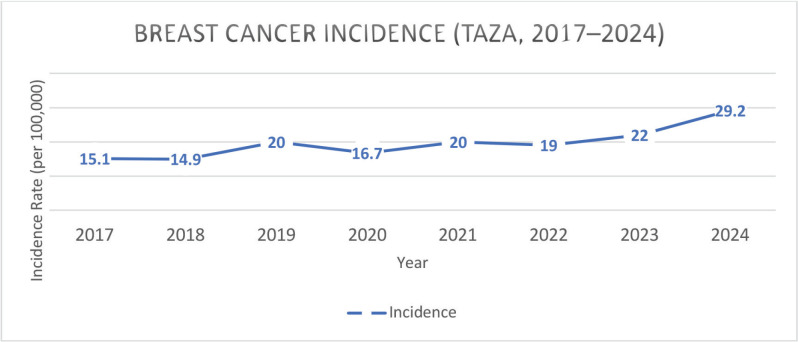
BC incidence in women (TAZA, 2017–2024).

Between November 2016 and December 2024, the RHRC of Taza Province recorded a total of 412 new BC cases among women, corresponding to an average of 52 cases per year. The recorded incidence rate was 15.1 per 100,000 in 2017 and remained nearly stable until 2018 (14.9). It increased to 20 per 100,000 in 2019, then fluctuated slightly between 2020 and 2022 (16.7 to 19.0), before rising again to 22.4 per 100,000 in 2023 and 29.2 per 100,000 in 2024. For analytical consistency, the temporal analysis of incidence was carried out from 2017 onwards, the centre's first full year of operation, while cases from 2016 were only included in the cumulative total. The eight cases recorded in 2016, which corresponded to a truncated period of observation (i.e., November to December), were included in the overall count (*n* = 412) but excluded from the calculation of annual incidence rates, as 2016 did not cover a full observation period (Figure 1).

This figure shows the annual incidence rates (per 100,000 women) of BC recorded at the Taza RHRC between 2017 and 2024. Denominators were based on the projected female population according to Haut-Commissariat au Plan (HCP) [11] data.

### Socio-economic characteristics of patients

The study population consisted of 412 patients with BC. The mean age at diagnosis was 51.5 ± 11.8 years, with extremes ranging from 23 to 90 years. Age distribution showed that 59% of patients were aged between 40 and 59, 25.2% were aged 60 or over and 15.9% were aged under 40 years, i.e., those classified as EOBC (Table 1).

In terms of marital status, 70.9% of patients were married. More than half of the patients 53% resided in rural areas. Overall, 73.5% of patients had health insurance coverage, while 26.5% had no coverage. Among insured patients, 40% were affiliated with RAMED, 13% with AMO-Tadamon, 15% with CNSS and 4.4% with CNOPS. Regarding employment status, 97.3% of patients reported no salaried employment (Table 1).

### Clinic and histologic characteristics of BC

Several features were noted regarding the clinical and anatomopathological characteristics of BC in our study of 412 patients (Table 2). The left breast was affected in 54.1% of patients, compared with 44.7% for the right breast. Bilateral involvement was observed in 1.2% of cases. Tumour localisation showed a predominance of the supero-external quadrant (SEQ), involved in 57.8% of cases. The supero-internal quadrant (SIQ) was involved in 20.4% of cases. Other localisations, notably the infero-external quadrant (IEQ) and infero-internal quadrant (IIQ) and the Retromammary region, were less frequent, each accounting for around 7%–8% of cases.

The main histological type was infiltrating ductal carcinoma, accounting for 95% of diagnoses. Other histological types included infiltrating lobular carcinoma (2%), mucinous (1%) and infiltrating papillary (1%).

Histoprognostic Scarff–Bloom–Richardson (SBR) grade analysis (Table 3) showed a predominance of grade II tumours, representing 68.4% of all cases. Among EOBC patients (<40 years), grades II and III accounted for 73.8% and 24.7% of cases, respectively, while grade I was rare (1.5%). Similarly, among non-EOBC patients (≥40 years), grade II represented 67.4% and grade III 28.2% of cases, with grade I remaining infrequent (4.3%). Overall, the distribution of SBR grades did not differ significantly between EOBC and non-EOBC groups (Fisher–Freeman–Halton exact test, *p* = 0.547).

## Discussion

Between November 2016 and December 2024, the RHRC of Taza recorded 412 newly diagnosed BC cases in women. Analysis of the trend shows a progressive increase in incidence, rising from 15 per 100,000 women in 2017 to 29 per 100,000 in 2024. The temporary decrease observed in 2020 aligns with the disruptions caused by the COVID-19 pandemic, which reduced access to routine screening and delayed non-urgent consultations, a pattern widely reported internationally [12].

From 2021 onward, the upward trend appears closely linked to a diagnostic catch-up effect combined with structural improvements in the province’s healthcare system. In particular, the opening of a private anatomical pathology laboratory in 2023 reduced diagnostic delays and increased the number of biopsy confirmations. The expansion of the AMO Tadamon insurance scheme [13] facilitated access to mammography and histological diagnosis for previously underserved rural women. Additionally, screening activities supported by the Lalla Salma Foundation, including the major mammography campaign of June 2024, temporarily increased case detection.

Overall, the rising local incidence mirrors national patterns, as documented by the Casablanca Cancer Registry, which reports an incidence rate of 52.6 per 100,000 women and a continuous upward trend since the early 2000s [7].

Comparable regional trends are observed across North Africa, where BC incidence reaches 45.4 per 100,000 women in Tunisia (2018) [14] and 61.9 per 100,000 in Algeria (2022) [15]. In contrast, high-income countries report substantially higher incidence values, with approximately 99 per 100,000 women in France [16] and 151 to 197 per 100,000 women in England according to national cancer registration statistics [17]. The gap likely reflects differences in screening coverage, age-structure of the female population, lifestyle risk profiles and cancer-registration capacity [18–20].

Taken together, these findings suggest that the increase observed in Taza reflects both enhanced diagnostic coverage and a broader epidemiological transition marked by changes in lifestyle-related risk factors [7, 21, 22]. It highlights the need to strengthen decentralised screening programs, reinforce community-based awareness and sustain local diagnostic capacities.

Our study shows an average age at diagnosis of 51.51 ± 11.8 years, with extremes of 23 and 90 years. The distribution of the number of patients by age shows that the highest numbers belong to the age group targeted by the BC screening programme in our country (40–69 years). This finding corroborates that of El Fouhi et al [9], which suggests that the average age of BC diagnoses at the Ibn Rochd University Hospital in Casablanca, Morocco was 51.6 years. This result is also similar to those observed in other neighboring countries, such as Tunisia [23] and Algeria [24]. This age profile reflects the usual epidemiological trend, in which the incidence of BC increases with age, particularly from the age of 50 onwards. Nevertheless, in our cohort, EOBC accounted for 15.8% of cases (Table 3), slightly lower than the 22% reported in the Casablanca Cancer Registry [7], This subgroup displayed higher-grade (SBR III) tumours, consistent with reports from Tunisia (14%–15%) [23] and Algeria (18%–19%) [24], where early-onset cases similarly show more aggressive tumour profiles, later clinical presentation and diagnostic delays in younger women [25]. At the other end of the spectrum, our study also reveals a relatively low proportion of cases in women over 70. This finding should not be interpreted as a low risk in this population, but could rather reflect limited access to screening, an underestimated diagnosis or a lesser use of the healthcare system by elderly women [26].

Married women constitute the majority of women diagnosed with BC, a pattern that primarily reflects the demographic structure of the Moroccan female population rather than any causal effect of marital status. National demographic data from the HCP indicate that marriage is the predominant marital status among adult women [27] – especially within the age groups most affected by BC – resulting in the expected over-representation of married women among diagnosed cases. Variations across marital-status groups may also reflect potential disparities in access to preventive services and screening [28]. Comparable observations have been reported in population-based studies from similar African countries [29, 30].

Our study shows a predominance of patients of rural origin compared with those from urban areas. This can be explained by the predominance of rural women in our region. Similar results were found in Tunisia by Mahjoub et al [23]. A more detailed analysis of the distance to the diagnostic center shows that almost 30% of patients live more than 30 km from the health center, while only 48.1% live less than 5 km away. This distance is a major factor in delaying diagnosis. Numerous studies [31, 32] have shown that geographical distance from screening facilities is associated with later consultation, a more advanced stage of the disease and poorer survival.

The distribution of patients according to their health cover reveals two main groups: on the one hand, those with health insurance, representing the majority of our sample, and on the other, those not covered by a social protection scheme who pay directly for their care, which may constitute an obstacle to access to screening and diagnostic services, particularly for the most vulnerable populations. The results obtained in our study diverge widely from those revealed by the Elkhalloufi et al study [32] at the National Institute of Oncology in Rabat (Morocco), which found that 77.1% of patients had no health insurance, which may be due to the generalisation of compulsory health insurance to the Moroccan population [33–35]. The type of health insurance, of which RAMED accounts for almost half, has a significant influence on how BC is detected and treated. A study in Turkey indicated that private insurance facilitates quicker and easier access to comprehensive care. On the other hand, those who are only covered by public health insurance face complicated pathways and inequalities in terms of care [36]. Similarly, in the United States, Medicare patients (public health insurance managed by the US government) receive biopsies and are treated earlier than those covered by commercial insurance, which highlights the advantages of certain types of insurance [37].

In our study, the vast majority of BC cases occurred among women who reported no salaried employment. This proportion is higher than that reported by Pranjić et al [38], who found that 52% of BC patients were not employed. The low frequency of salaried women diagnosed at our center may partly reflect the relatively high female unemployment rate in Morocco (16.8% in 2021) [39]. In addition, salaried women may preferentially seek diagnosis and care in the private sector, which could contribute to an under-representation of these patients in our hospital-based series [40, 41].

For the localisation of the tumour in the patients in our study. Our results are consistent with the study by Mahjoub et al [23], which revealed that in 54% of cases, the tumours were located in the left breast, 45% in the right breast, while in 2% of cases, the tumours were bilateral.

In terms of tumour localisation, the SEQ is the most frequently affected area, followed by the SIQ. Other localisations, such as the IEQ and IIQ, as well as the retromammary zone, are affected more marginally. These observations are consistent with the results reported in several previous studies, which also highlight the predominance of the SEQ as the main site of breast lesions. El Fouhi et al [9] and Mahjoub et al [23], due to the excessive amount of glandular tissue in the central and superolateral part of the breast [42].

In our study, as in the literature, infiltrating ductal carcinoma stands out as the most common histological type of BC, accounting for a large majority of diagnosed cases. Conversely, less common forms, such as infiltrating lobular carcinoma and rare variants such as mucinous carcinoma and infiltrating papillary carcinoma, are seen in only a minority of cases. This predominance of invasive ductal carcinoma is also confirmed by several previous studies [9, 23, 43].

The results of our study show that most tumours were classified as SBR grades II and III, with grade II being the most frequent (Table 3). This pattern is consistent with the literature, where grade II is commonly reported as the predominant category [9, 44, 45]. Several studies [8, 42] have suggested that BC in younger women tends to be more aggressive, with a higher proportion of SBR grade III tumours. In our study, 15.8% of patients were younger than 40 years (EOBC) and grade III accounted for 24.7% of cases in this group. However, the distribution of SBR grades did not differ significantly between EOBC and non-EOBC patients (Fisher–Freeman–Halton exact test, p = 0.547), indicating that histological grade was comparable across age groups in our population.

In summary, the left breast was more frequently affected, with a predominance of tumours located in the upper-external quadrant, in line with classical findings from the literature. Invasive ductal carcinoma was the most common histological type. Overall, grades II and III predominated, reflecting a substantial proportion of moderate- to high-grade tumours in the study population, without evidence of a significant age-related difference in SBR grade.

## Conclusion

BC incidence in Taza Province has increased steadily between 2017 and 2024, likely reflecting improved diagnostic coverage as well as changing lifestyle-related risk factors. Early-onset cases (<40 years) account for 15.8% and are characterised by higher-grade tumours, underscoring the need for tailored awareness and early detection programs targeting younger women. Strengthening decentralised screening, ensuring equitable access to mammography and supporting regional pathology services remain essential to reduce diagnostic delays.

This study benefits from an 8-year dataset and a multidimensional epidemiological–clinical–histopathological approach, providing rare insights into a largely undocumented and predominantly rural population. However, potential selection bias due to private-sector diagnoses and the lack of systematic follow-up – particularly treatment and survival outcome data – may limit the completeness of regional estimates and preclude survival analyses (e.g., comparisons between EOBC and non-EOBC), thereby constraining clinical interpretation.

Overall, these findings highlight the urgent need to reinforce early detection, improve access to specialised care and adapt BC control strategies to the socioeconomic and geographic realities of eastern Morocco.

## List of abbreviations

AMO, Assurance Maladie Obligatoire (Compulsory Health Insurance); BC, Breast cancer; CNOPS: Caisse Nationale des Organismes de Prévoyance Sociale (National Social Security Fund); CNSS, Caisse Nationale de Sécurité Sociale (National Social Security Fund); EOBC, Early onset breast cancer; IEQ, Infero-external quadrant; IIQ: Infero-internal quadrant; SBR, Scarff-Bloom-Richardson cited; SEQ, Supero-external quadrant; SIQ, Supero-internal quadrant; RAMED: Régime d’Assistance Médicale pour les personnes Économiquement Démunies (Medical Assistance Scheme for the Economically Disadvantaged); RHRC, Reproductive Health Reference Center.

## Conflicts of interest

None to disclose.

## Funding

This research did not receive any financial support from external sources.

## Author contributions

NB (Concept, Design, Resources, Materials, Data Collection and Processing, Analysis and Interpretation, Literature Search, Writing Manuscript), EE (Concept, Design, Supervision, Critical review), FE (Concept, Design, Literature Search), KH (Analysis and Interpretation, Literature Search). All authors read and approved the final version of the manuscript.

## Figures and Tables

**Table 1.** Socio-demographic characteristics of women.

Variables		(n)	(%)
Age	20 to 39 years	65	15.8
	40 to 59 years	243	59
	60 years and older	104	25.2
Marital status	Married	292	70.9
	Divorced	23	5.6
	Widowed	36	8.7
	Single	61	14.8
Residence	Rural	216	52.6
	Urban	195	47.4
	Less than 5 km	198	48
Distance from home to RHRC	From 5 to 15 km	44	10.7
	From 16 to 30 km	48	11.7
	More than 30 km	122	29.6
Salaried	No	401	97.3
	Yes	11	2.7
Health insurance	No	109	26.5
	Yes	303	73.5
	CNSS	62	15
	CNOPS	18	4.4
Type of health insurance	RAMED	166	40.3
	AMO Tadamone	57	13.8
	No	109	26.5

**Table 2.** Clinic and histologic characteristics of BC.

Variables		(n)	(%)
	Right breast	184	44.7
Cancer site	Left breast	223	54.1
	Bilateral	5	1.2
Localization	SEQ	238	57.8
	SIQ	84	20.4
	IEQ	33	8
	IIQ	28	6.8
	Retromammary	29	7
Cancer type	Infiltrating ductal carcinoma	391	94.8
	Infiltrating lobular carcinoma	7	1.7
	Infiltrating mucinous carcinoma	4	1
	Infiltrating papillary carcinoma	4	1
	Ductal carcinoma in situ	6	1.5
	Grade I	16	3.9
SBR grade	Grade II	282	68.4
	Grade III	114	27.7

**Table 3.** Distribution of SBR histoprognostic grade by EOBC status.

Age group	SBR grade I, n (%)	SBR grade II, n (%)	SBR grade III, n (%)	Total, n (%)
EOBC (<40 years)	1 (1.5)	48 (73.8)	16 (24.7)	65 (15.8)
Non-EOBC (≥40 years)	15 (4.3)	234 (67.4)	98 (28.2)	347 (84.2)
Total	16 (3.9)	282 (68.4)	114 (27.7)	412 (100)

Note: Percentages for SBR grades are row percentages. Total column percentages are calculated over the full sample (*N* = 412) Statistical test: Fisher–Freeman–Halton exact test (two-sided), *p* = 0.547
